# Supplementary material for: Model evidence for a seasonal bias in Antarctic ice cores
Source: Nat Commun. 2018 Apr 10;9:1361. doi: 10.1038/s41467-018-03800-0 (PMC5893564; doi:10.1038/s41467-018-03800-0)
Supplement: Supplementary file 1 — Supplementary Information [file 41467_2018_3800_MOESM1_ESM.pdf]

Model evidence for a seasonal bias in Antarctic ice cores

Erb et al.

Supplementary Materials

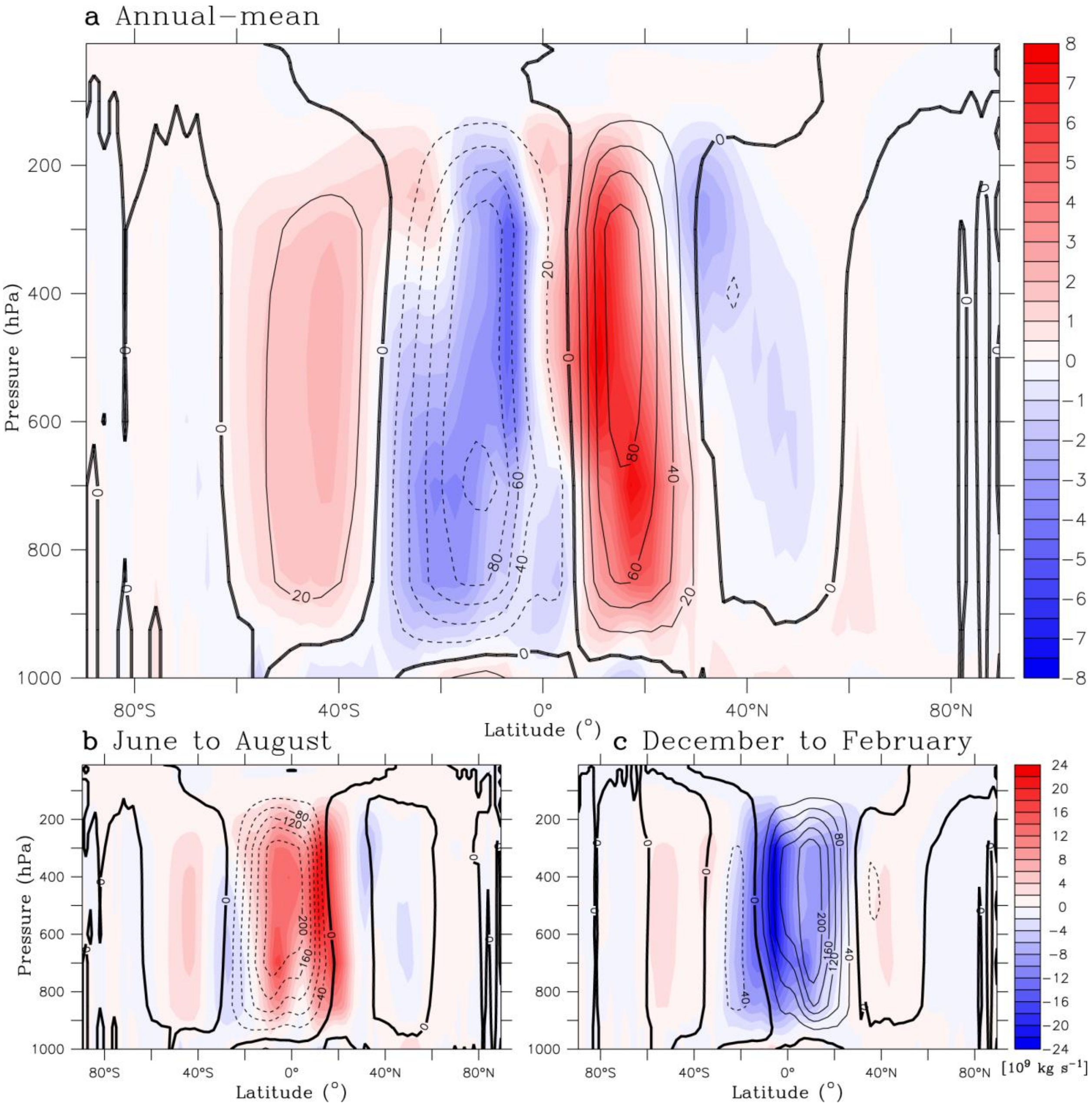

Change in atmospheric overturning in response to lowered obliquity

Supplementary Figure 1. Change in zonal-mean atmospheric overturning stream-function (colors, 10^9 kg s^{-1}) in the CM2.1 low – high obliquity experiment for (a) annual-mean, (b) June to August, and (c) December to February, similar to [1]. Contours show

the high obliquity overturning stream-function. The apparent strengthening of the Hadley Circulation in the annual-mean can be better understood as the result of seasonal changes: a weakening of the winter-hemisphere Hadley cell, strengthening of the summer-hemisphere Hadley cell, and an equatorward shift of the rising branch.

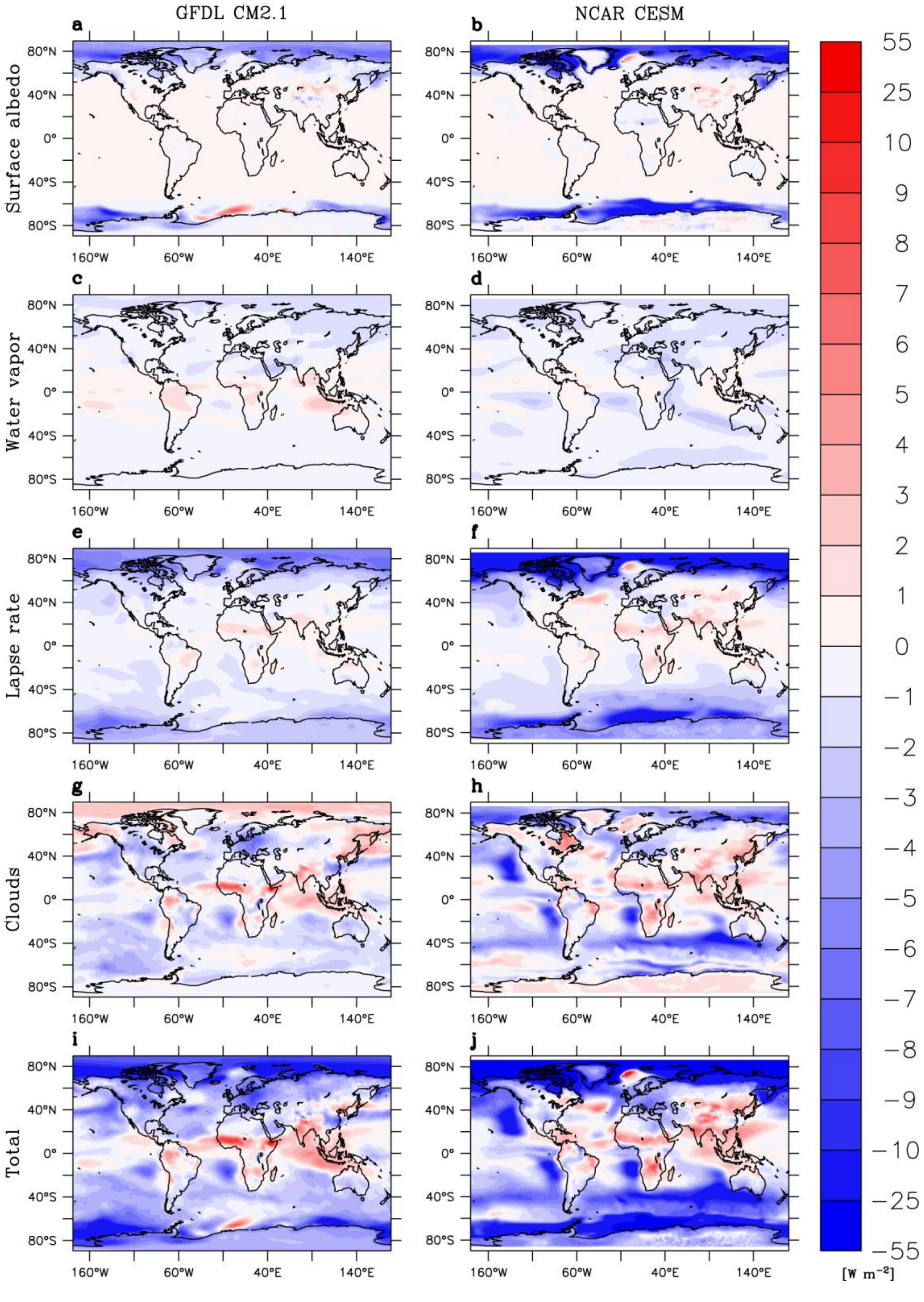

Effect of radiative feedbacks in response to lowered obliquity

Supplementary Figure 2. The effect of radiative feedbacks on net top of atmosphere radiation (W m^{-2}) in response to lowered obliquity in CM2.1 (left) and CESM (right). Positive values represent an increase in net downward top of atmosphere radiation. From top to bottom, the effects of feedbacks are calculated for surface albedo, water vapor, lapse rate, clouds, and the total, which is a sum of the previous four. Calculations were based on radiative kernels computed with AM2 (for CM2.1) and CAM3 (for CESM), although radiative kernels for different models are relatively similar[2].

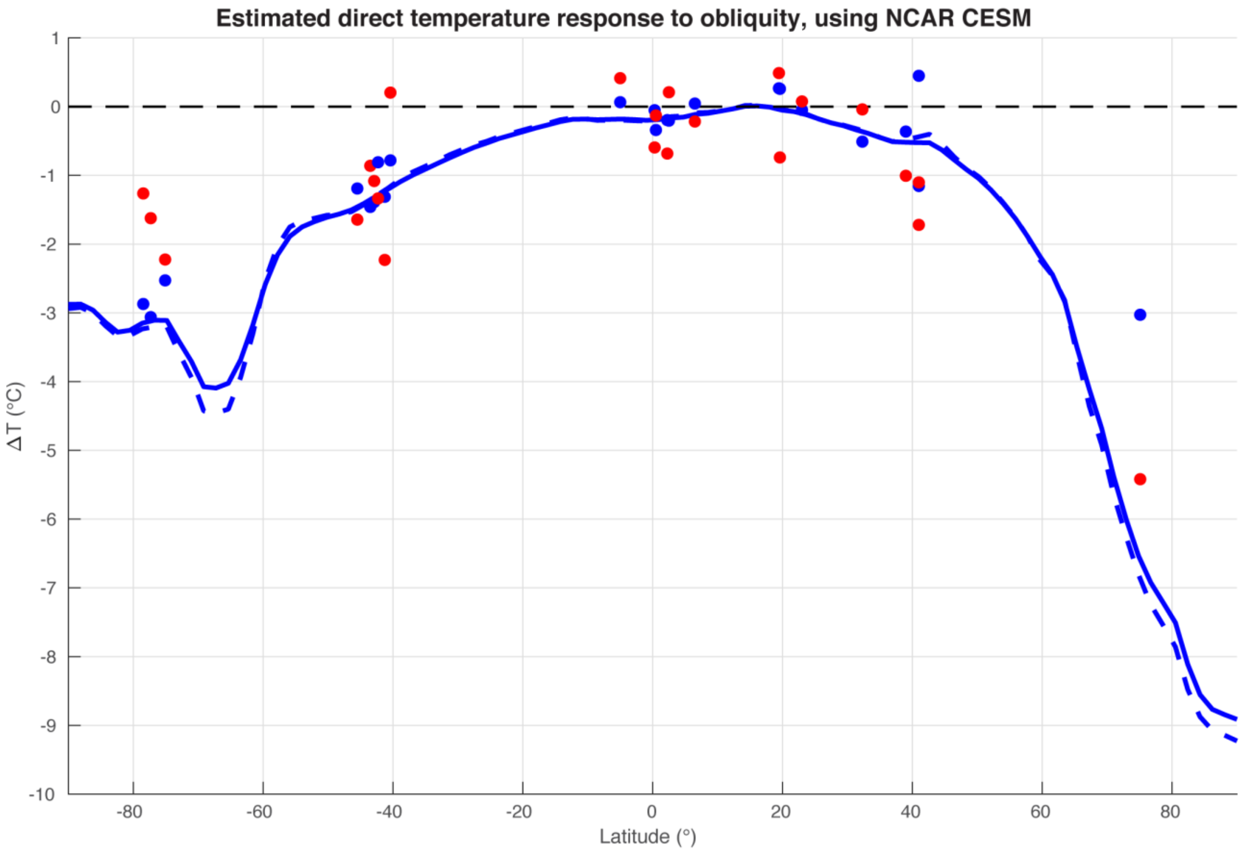

Obliquity temperature response in NCAR CESM and estimated from proxies

Supplementary Figure 3. Like Fig. 3 in the main text, but for CESM. Temperature changes at specific proxy locations in the CESM low – high obliquity experiment (blue dots) and the apparent direct short-term obliquity response in proxies (red dots), which were derived by scaling the modeled obliquity response in the linear reconstructions to match the proxies. To show how the modeled temperature anomalies at proxy locations compare to zonal-mean values, lines show the zonal-mean anomalies for both surface air temperature (solid) and surface temperature (dashed) in CESM. The red dots shown here differ from those in Fig. 3 because the obliquity response in proxies is determined through comparison with the model-based reconstructions. Because the

precession, greenhouse gas, and ice sheet components of the reconstructions differ between models, the proxy variations unaccounted for by those factors also differ, so the estimation of the short-term obliquity response in proxies is somewhat different.

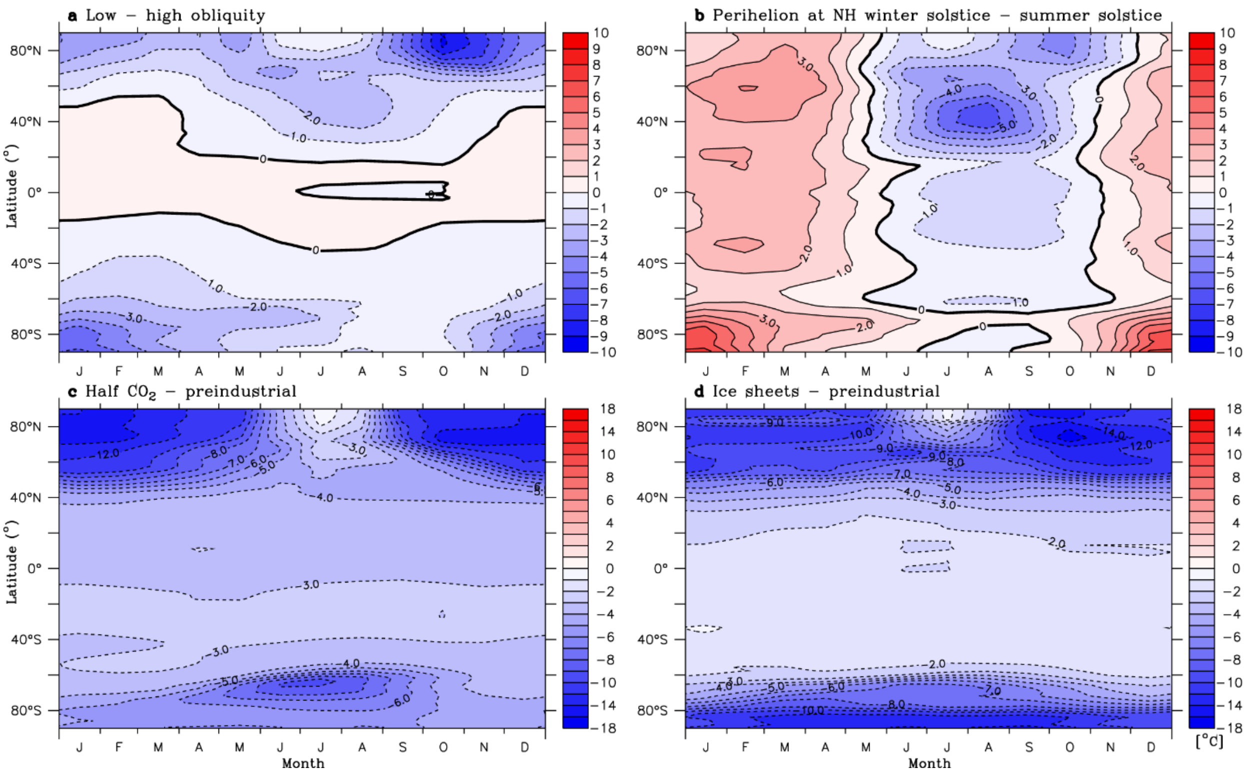

Modeled zonal-mean temperature response to different forcings

Supplementary Figure 4. Zonal-mean surface air temperature anomalies in four CM2.1 single-forcing "fingerprint" experiments: (a) low – high obliquity, (b) perihelion at Northern Hemisphere winter solstice – summer solstice, (c) half CO_2 – preindustrial, and (d) ice sheets – preindustrial. The linear reconstruction makes use of two equinox simulations and a zero eccentricity simulation as well, but (b) only shows the temperature anomaly between solstice states for simplicity.

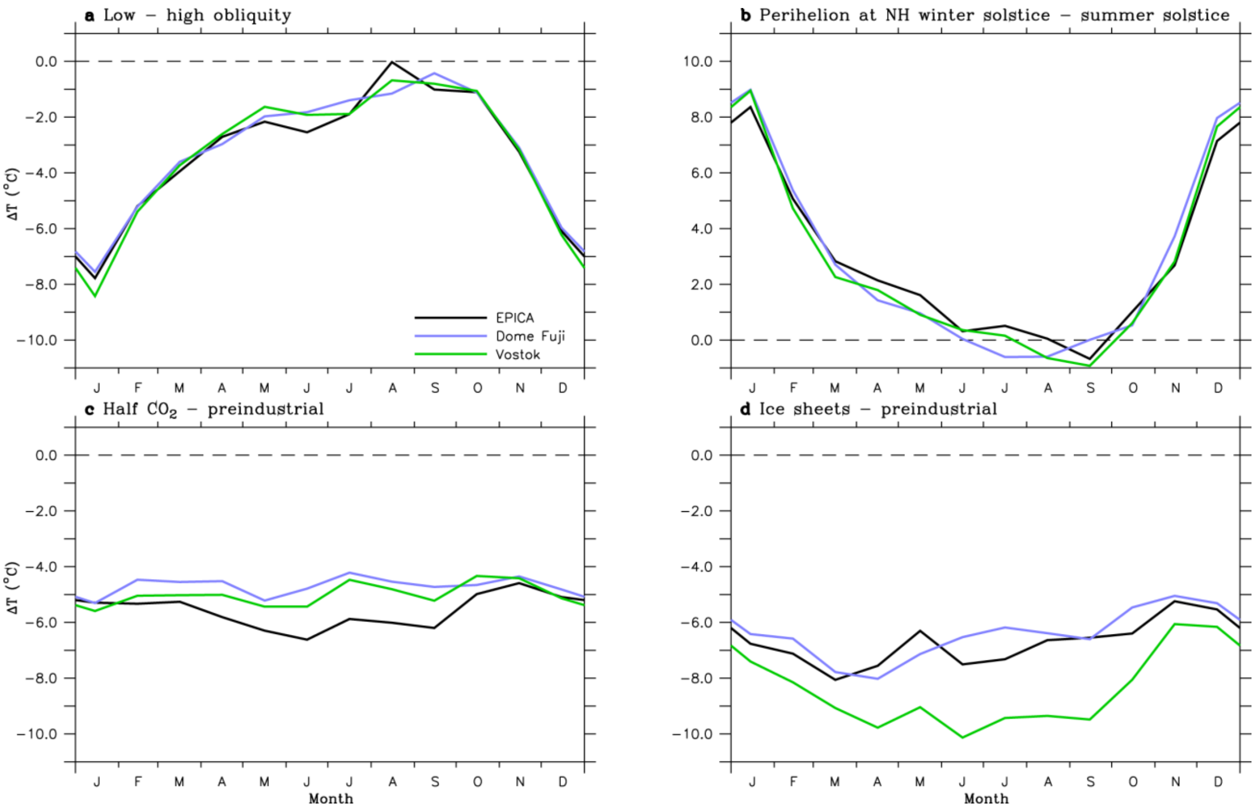

Modeled temperature response to difference forcings at ice core locations

Supplementary Figure 5. Surface air temperature anomalies at the location of Antarctic ice cores in four CM2.1 single-forcing “fingerprint” experiments: (a) low – high obliquity, (b) perihelion at Northern Hemisphere winter solstice – summer solstice, (c) half CO_2 – preindustrial, and (d) ice sheets – preindustrial. Lines correspond with the locations of the EPICA Dome C (black), Dome Fuji (blue), and Vostok (green) ice cores. The linear reconstruction makes use of two equinox simulations and a zero eccentricity simulation as well, but (b) only shows the temperature anomaly between solstice states for simplicity. The temperature responses to obliquity and precession vary considerably over the course of the year at these locations, while the responses to CO_2 and ice sheets are more temporally uniform.

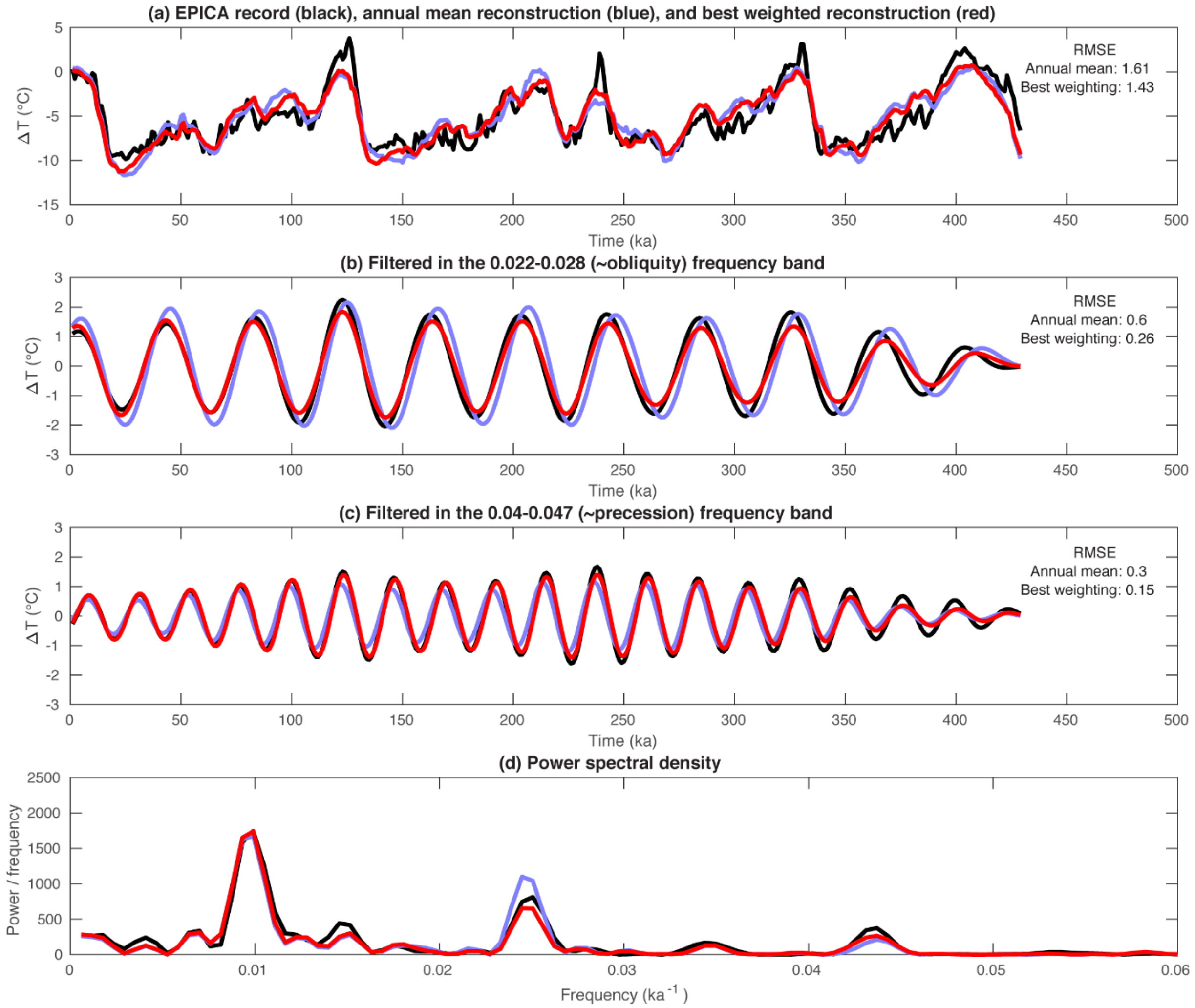

Analysis of temperature signals for EPICA Dome C and linear reconstruction

Supplementary Figure 6. Analysis of the EPICA Dome C proxy temperature record (black), unfitted annual-mean linear reconstruction (blue), and the best-fit seasonally-weighted linear reconstruction (red) at that location. The linear reconstructions use the GFDL CM2.1 model, and the best-fit seasonally-weighted linear reconstruction (red) is chosen based on the results shown in Fig. 4 in the main text. (a) Proxy temperature record and reconstructions. (b,c) Records filtered in the obliquity and precession bands, respectively, using a butterworth filter, with root mean square error (RMSE) calculated

between each of the reconstructions and the proxy signal. (d) Lomb-Scargle power spectral density. This figure helps visualize both the magnitude and phasing of the obliquity and precession components of the EPICA Dome C proxy record and reconstructions.

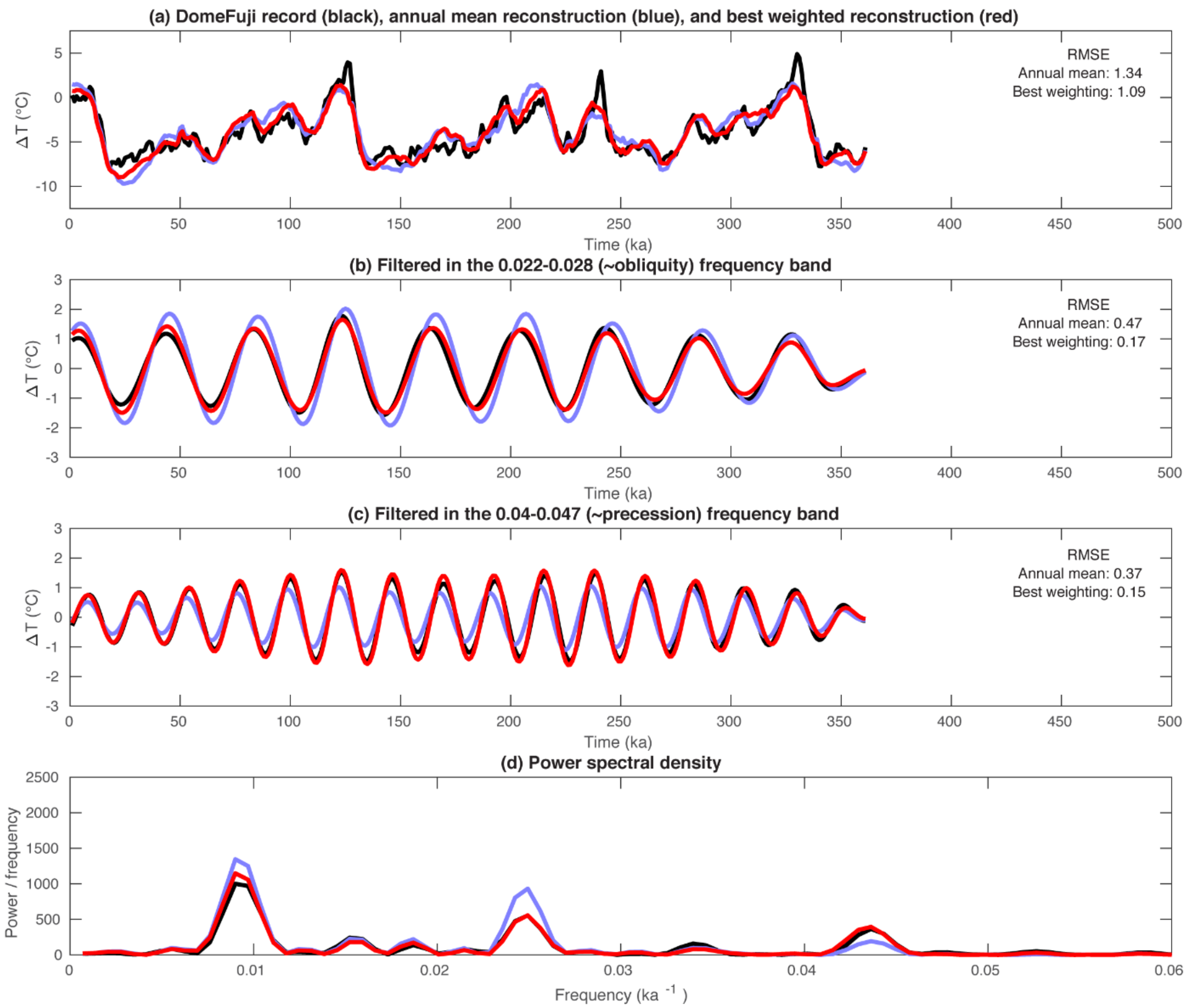

Analysis of temperature signals for Dome Fuji and linear reconstruction

Supplementary Figure 7. Analysis of the Dome Fuji proxy temperature record (black), unfitted annual-mean linear reconstruction (blue), and the best-fit seasonally-weighted linear reconstruction (red) at that location. The linear reconstructions use the GFDL CM2.1 model, and the best-fit seasonally-weighted linear reconstruction (red) is chosen based on the results shown in Fig. 4 in the main text. (a) Proxy temperature record and reconstructions. (b,c) Records filtered in the obliquity and precession bands, respectively, using a butterworth filter, with root mean square error (RMSE) calculated

between each of the reconstructions and the proxy signal. (d) Lomb-Scargle power spectral density. This figure helps visualize both the magnitude and phasing of the obliquity and precession components of the Dome Fuji proxy record and reconstructions.

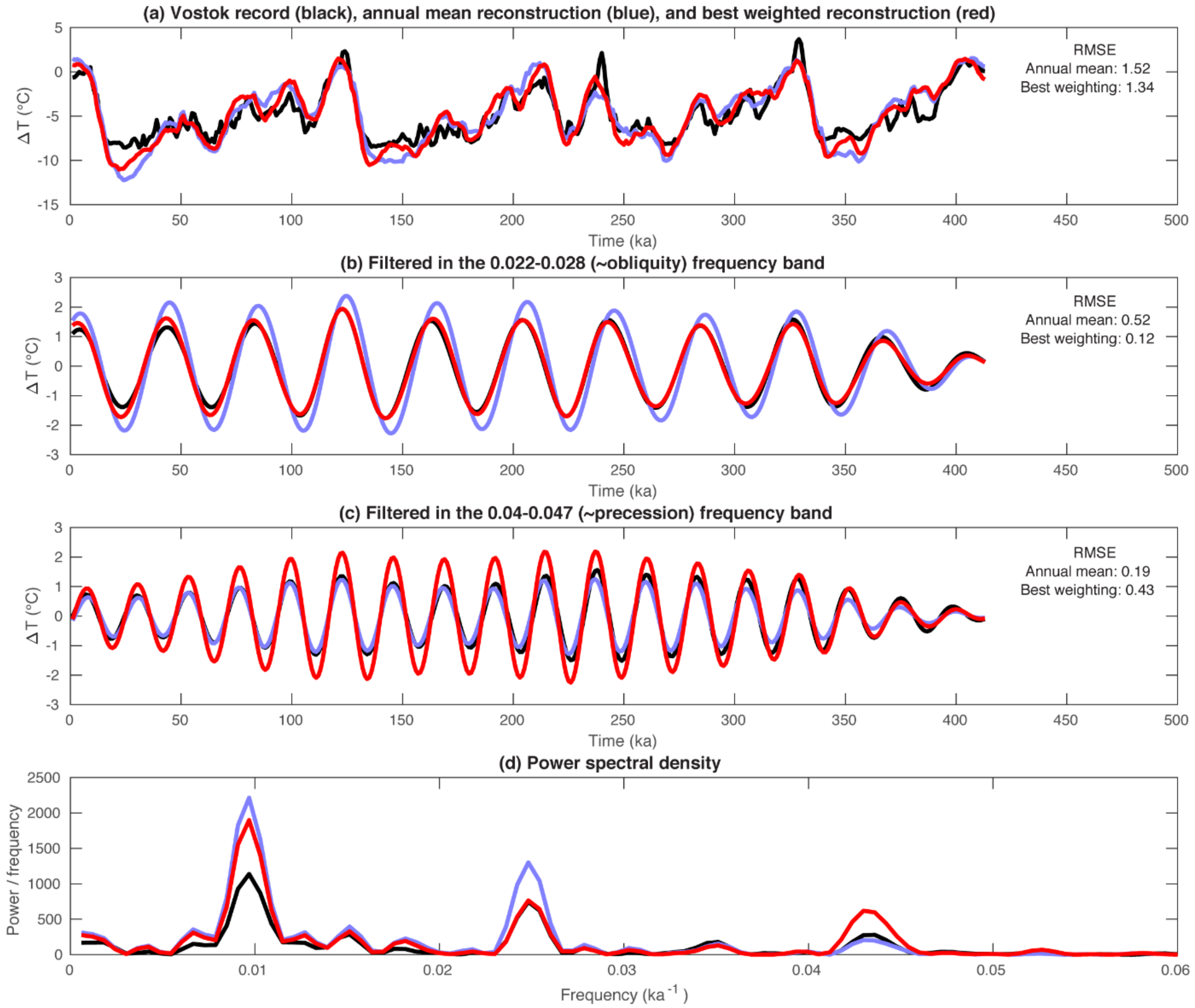

Analysis of temperature signals for Vostok and linear reconstruction

Supplementary Figure 8. Analysis of the Vostok proxy temperature record (black), unfitted annual-mean linear reconstruction (blue), and the best-fit seasonally-weighted linear reconstruction (red) at that location. The linear reconstructions use the GFDL CM2.1 model, and the best-fit seasonally-weighted linear reconstruction (red) is chosen based on the results shown in Fig. 4 in the main text. (a) Proxy temperature record and reconstructions. (b,c) Records filtered in the obliquity and precession bands, respectively, using a butterworth filter, with root mean square error (RMSE) calculated

between each of the reconstructions and the proxy signal. (d) Lomb-Scargle power spectral density. This figure helps visualize both the magnitude and phasing of the obliquity and precession components of the Vostok proxy record and reconstructions.

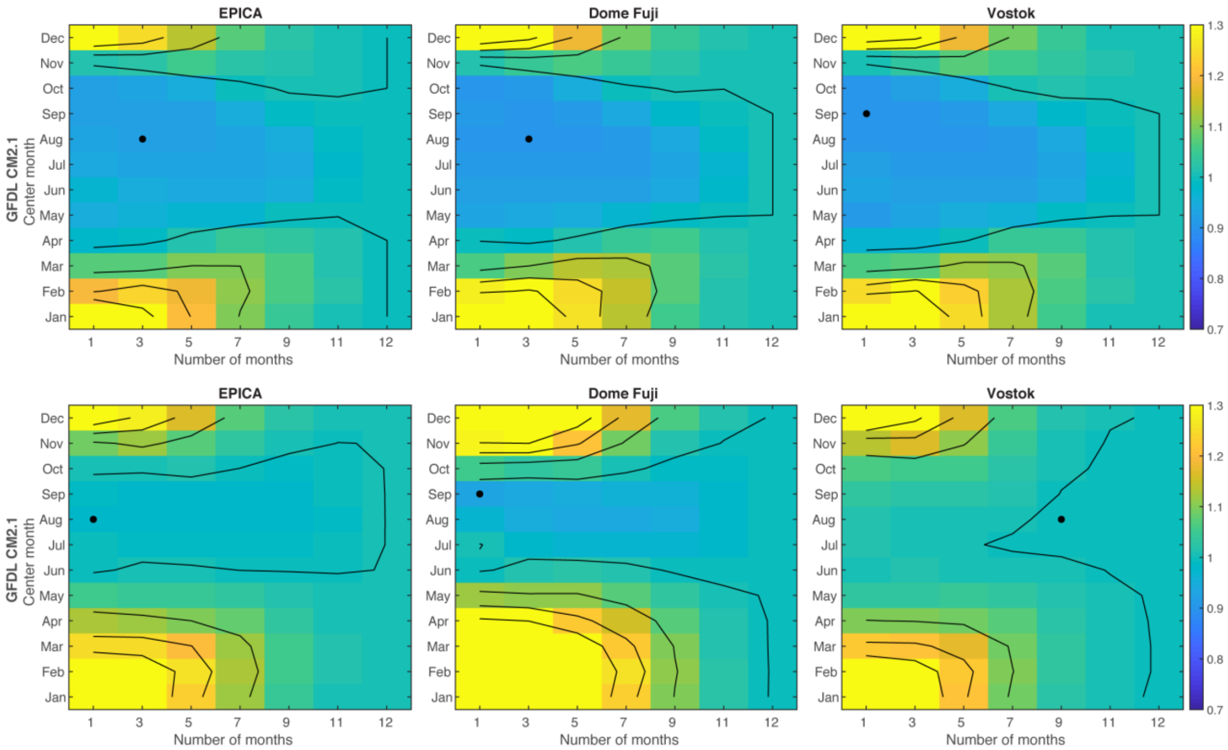

Mismatches when only obliquity or precession are affected by a monthly average, using GFDL CM2.1

Supplementary Figure 9. Relative mismatch between proxy temperature records and CM2.1 linear reconstructions using different monthly averages, where only specific components of the reconstruction are affected by the monthly average. Each grid box corresponds to the mismatch for a linear reconstruction where the obliquity term (top) or precession term (bottom) is averaged over a portion of the year. This figure is like the first row of Fig. 4 in the main text, but the choice of seasonal average only affects the obliquity term (top) or the precession term (bottom) for CM2.1. The y-axis defines the central month of the mean and the x-axis defines the number of months included in the mean. The rightmost column (labeled 12) indicates a 12 month mean. Other components of the reconstruction are calculated as annual-means. This experimental

design examines whether ice core obliquity and precession signals are more consistent with a seasonal signal than the annual-mean. Values are the root mean square error (RMSE) of each monthly-mean case normalized by the RMSE of the annual-mean case, so monthly-mean reconstructions which better match the proxy record compared to the annual-mean case have values <1. Contours are drawn from 0.7 to 1.3 with a contour interval of 0.1. The black dot indicates the lowest RMSE. The calculation is done for all three Antarctic locations (EPICA Dome C, Dome Fuji, and Vostok) for GFDL CM2.1 linear reconstructions.

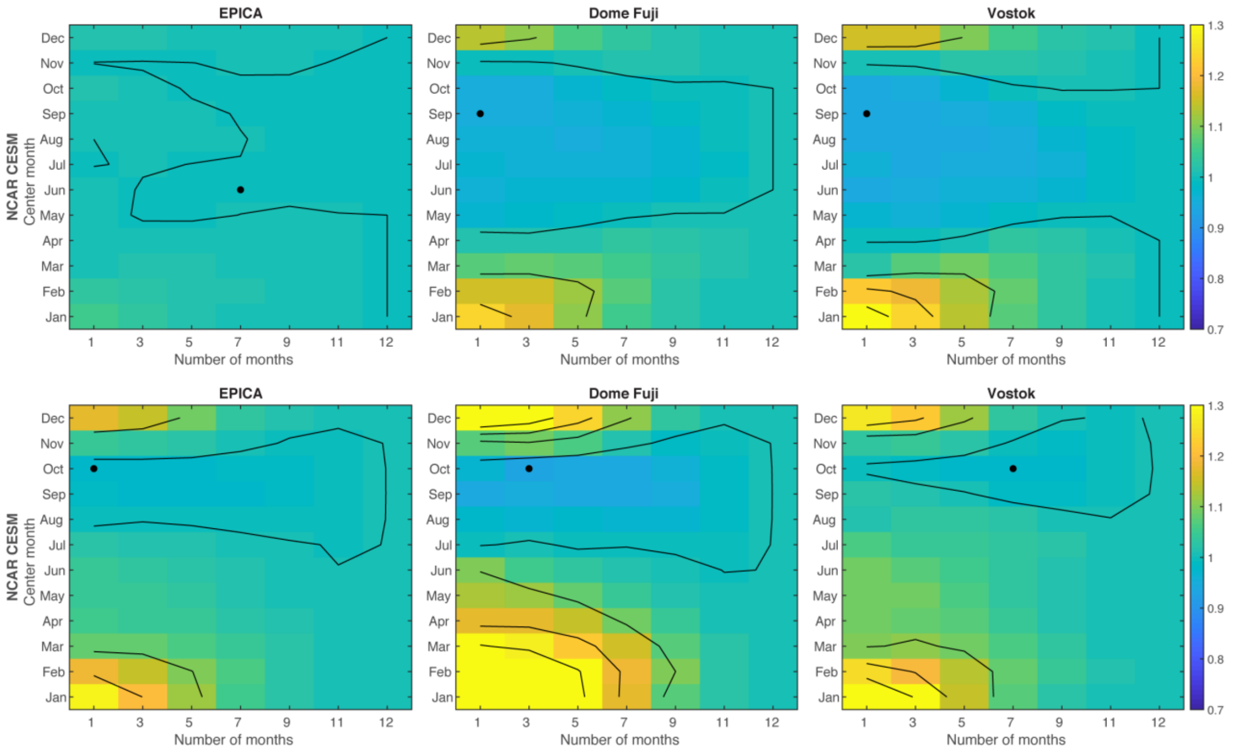

Mismatches when only obliquity or precession are affected by a monthly average, using NCAR CESM

Supplementary Figure 10. Relative mismatch between proxy temperature records and CESM linear reconstructions using different monthly averages, where only specific components of the reconstruction are affected by the monthly average. Each grid box corresponds to the mismatch for a linear reconstruction where the obliquity term (top) or precession term (bottom) is averaged over a portion of the year. This figure is like the second row of Fig. 4 in the main text, but the choice of seasonal average only affects the obliquity term (top) or the precession term (bottom) for CESM. The y-axis defines the central month of the mean and the x-axis defines the number of months included in the mean. The rightmost column (labeled 12) indicates a 12 month mean. Other components of the reconstruction are calculated as annual-means. This experimental

design examines whether ice core obliquity and precession signals are more consistent with a seasonal signal than the annual-mean. Values are the root mean square error (RMSE) of each monthly-mean case normalized by the RMSE of the annual-mean case, so monthly-mean reconstructions which better match the proxy record compared to the annual-mean case have values <1. Contours are drawn from 0.7 to 1.3 with a contour interval of 0.1. The black dot indicates the lowest RMSE. The calculation is done for all three Antarctic locations (EPICA Dome C, Dome Fuji, and Vostok) for NCAR CESM linear reconstructions.

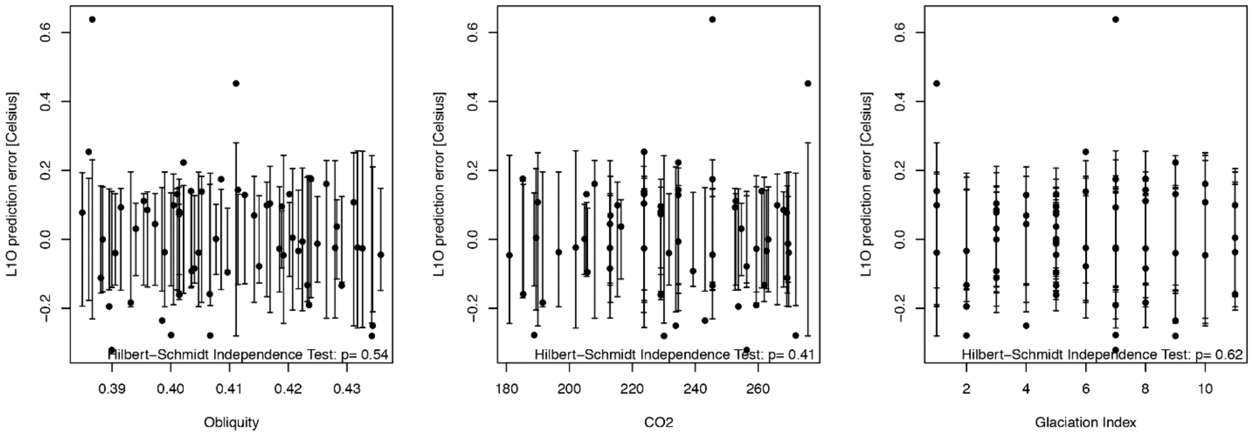

Prediction errors for the HadCM3 climate emulator

Supplementary Figure 11. Leave-one-out Gaussian process prediction errors, along with the standard deviations of these errors, as predicted by the Gaussian process emulator, plotted as a function of input variables. In other words: for each point, one simulation is left out of the emulator's construction and then estimated from the emulator, and the difference between mean Antarctic temperature in the actual simulation and in the estimate is plotted. The error for each omitted simulation is plotted, indicating the approximate range of errors over parameter space. The *p*-values based on the Hilbert Schmidt Independence Criterion are always well above 0.05, which does not allow us to reject the null-hypothesis that errors are independent of prediction errors.

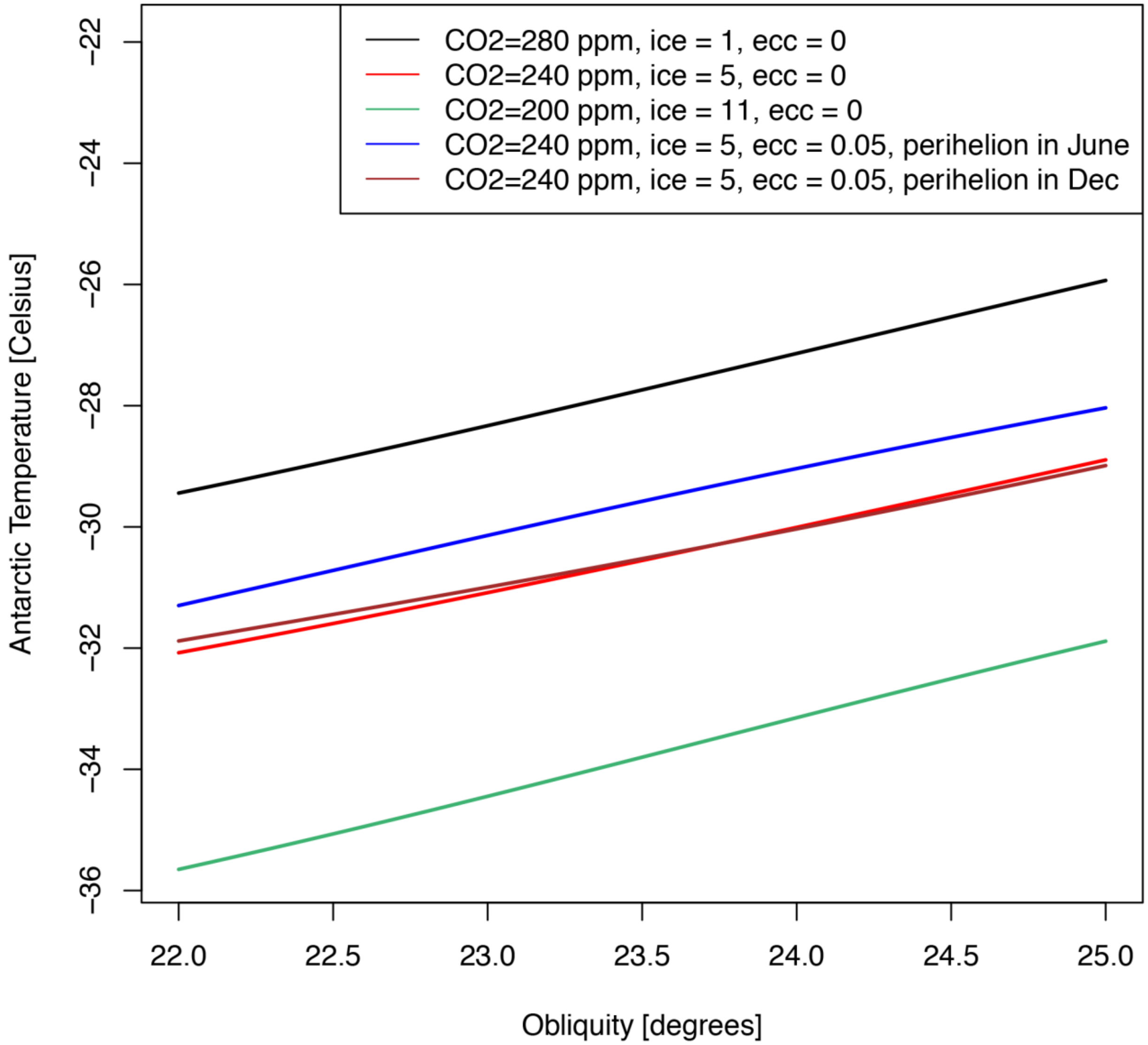

Antarctic temperature predicted by the HadCM3 climate emulator

Supplementary Figure 12. Annual-mean Antarctic temperature predicted by the HadCM3 emulator as a function of obliquity in different precession, CO_2, and glacial configurations. *Ecc* is "eccentricity", and *ice* is a glaciation index representing the degree of glaciation, equal to 1 at the Holocene and 11 at the Last Glacial Maximum.

Supplementary Tables

Forcing parameters					
	Obliquity (°)	Longitude of perihelion (°)	Eccentricity	CO_2 (ppm)	Ice sheets
Preindustrial	23.439 (23.44107)	102.93 (102.7242)	0.0167 (0.01670772)	286 (284.7)	0 ka BP
Low obliquity	22.079	---	---	---	---
High obliquity	24.480	---	---	---	---
AE perihelion	---	0	0.0493	---	---
WS perihelion	---	90	0.0493	---	---
VE perihelion	---	180	0.0493	---	---
SS perihelion	---	270	0.0493	---	---
Zero eccentricity	---	---	0	---	---
Half CO2	---	---	---	143 (142.35)	---
Ice sheets	---	---	---	---	21 ka BP

Forcings parameters for single-forcing simulations

Supplementary Table 1. Forcing parameters for the GFDL CM2.1 and NCAR CESM single-forcing simulations, as in [3]. Where CESM forcing parameters differ from those used in GFDL CM2.1, the CESM values are given in parentheses. These differences stem from slight differences in the preindustrial simulations. “---” indicates values

identical to the respective preindustrial simulations. Precession simulations have perihelion at the Northern Hemisphere autumnal equinox (AE), winter solstice (WS), vernal equinox (VE), and summer solstice (SS). Different ice sheet reconstructions are used in the ice sheet simulations, as details in the methods section.

Proxy records					
Name	Proxy type	Latitude	Longitude	Time period	Reference
NGRIP	$\delta^{15}N$	75.1°N	42.32°W	10-123 ka	[4]
U1313	Uk'37	41°N	33°W	327-957 ka	[5]
ODP 1020	Uk'37	41°N	126.4°W	4-810 ka	[6]
MD03-2699	Uk'37	39°N	10.7°W	301-580 ka	[7]
ODP 1012	Uk'37	32.3N	118.6°W	0-5070 ka	[8]
LPAZ21P	Uk'37	23°N	109.5°W	1-236 ka	[8]
ODP 1145	Mg/Ca	19.6°N	117.6°E	2-142 ka	[9]
ODP 1146	Uk'37	19.45°N	116.2°E	4-2160 ka	[10]
MD06-3067	Mg/Ca	6.5°N	126.5°E	5-158 ka	[11]
MD03-2707	Mg/Ca	2.5°N	9.4°E	0-155 ka	[12]
TR163-19	Mg/Ca	2.25°N	90.95°W	1-361 ka	[13]
TR163-22	Mg/Ca	0.5°N	92.4°W	1-134 ka	[14]
ODP 806	Mg/Ca	0.3°N	159.4°E	4-647 ka	[15]
GeoB10038	Mg/Ca	5°S	103°E	0-131 ka	[16]
MD97-2121	Uk'37	40.4°S	178°E	3-135 ka	[17]
PS2495-3	Mg/Ca	41.27°S	14.49°W	2-153 ka	[18]
SO136-GC3	Uk'37	42.3°S	169.9°E	4-287 ka	[19]
ODP 1090	Uk'37	42.91°S	8.9°E	0-3636 ka	[20]
RC11-120	Mg/Ca	43.5°S	79.9°E	3-294 ka	[21]
MD97-2120	Mg/Ca	45.5°S	174.9°E	2-336 ka	[22]

EPICA Dome C	δD	75.1°S	123.35°E	1-430 ka	[23]
Dome Fuji	δD and $\delta^{18}O$	77.32°S	39.7°E	1-361 ka	[24]
Vostok	δD	78.47°S	106.8°E	1-413 ka	[25]

Information about proxy records

Supplementary Table 2. Information about proxy records used the present research, sorted from north to south. All sediment cores were compiled and processed in past work[26], while the ice cores (NGRIP, EPICA Dome C, Dome Fuji, and Vostok) were retrieved from other sources. The “time period” gives the entire length of the record, but the total linear reconstruction only covers 1-430 ka, so data outside of that timespan has no effect on the comparison.

Supplementary Notes

Supplementary Note 1: The response of the Hadley Circulation to changes in obliquity

At present, the Hadley circulation has a pronounced seasonal cycle consisting of a strong winter-hemisphere Hadley cell and a weaker summer-hemisphere Hadley cell[1,27]. At low obliquity, the latitude of maximum insolation and consequently the rising branch of the Hadley circulation remains closer to the equator (Supplementary Fig. 1). This has two effects. First, the winter Hadley cell weakens, reducing cross-equatorial heat transport[1]. Second, the summer Hadley cell strengthens, increasing transport of potential energy from near the equator toward the extra-tropics in CESM (although most of this is counteracted by opposite changes in sensible and latent heat transport). This helps explain the enhanced poleward heat transport in the tropics described in the main text.

Supplementary Note 2: Radiative feedbacks

To better understand the temperature response to lowered obliquity, the effects of surface albedo, water vapor, lapse rate, and cloud feedbacks on net top of atmosphere radiation are calculated from the CM2.1 and CESM experiments using the radiative kernel method[2,28]. In the polar regions, surface albedo and lapse rate feedbacks play the dominant role (Supplementary Fig. 2). During low obliquity, Arctic sea ice maintains a much larger area in Northern Hemisphere summer and fall and

Antarctic sea ice has a larger area throughout much of the year, both of which increase surface albedo. Additionally, high-latitude cooling occurs most strongly in the lower troposphere, helping maintain colder temperatures through the lapse rate feedback. Because the upper atmosphere emits more of the radiation that escapes to space, and thus is more important to radiative balance, additional cooling can occur at lower altitudes without affecting the radiative balance as greatly. Slow feedbacks, such as changes in ice sheets or atmospheric composition, are not present in these experiments.

Supplementary Note 3: Spectral analysis

Age models present a source of uncertainty in the present analysis. If ages in the temperature and forcing time series are not consistent, the magnitude of direct short term obliquity response estimated in the proxy records, which should be concurrent with the obliquity forcing, may appear to be smaller or larger than it actually is. To test whether an artificial phasing offset due to age model uncertainties presents a problem in the current paper, a spectral analysis has been computed for the GFDL CM2.1-based linear reconstructions. Antarctic records and model-based reconstructions have been filtered in the obliquity and precession bands (Supplementary Figs. 6-8). Note that the proxy records combine the direct short-term response to orbital insolation forcing and the slower response associated with ice sheet and carbon cycle feedbacks. In the linear reconstruction, an obliquity signal exists in the sea level record, lagged from the direct obliquity signal by ~10 ka. This lagged obliquity signal is consistent with a

delayed response of ice sheets. The ability of obliquity to modify Antarctic ice is suggested, for example, in Antarctic debris-covered glaciers[29].

For Dome Fuji and Vostok (although not always for EPICA Dome C), the obliquity variations in the annual-mean reconstruction are larger than those in proxy records, even when timing of the events is ignored. In comparison, the monthly-weighted reconstruction (red curves in Supplementary Figs. 6-8) better matches many of the signals.

Because of the seasonal nature of the obliquity forcing, a seasonal average of the climate response can either increase or decrease the apparent magnitude of the direct climate response to obliquity. However, the obliquity signal present in the sea level record, slightly lagged from the direct forcing, is largely unchanged because the climate response to ice sheets is less seasonal (Supplementary Figs. 4,5). Since the seasonal weighting reduces the apparent direct climate response but leaves the lagged response relatively unchanged, the net obliquity signal is reduced in amplitude and shifted in time toward the present, in better agreement with the proxy records. For all three Antarctic ice cores, the obliquity signal is better matched in the seasonally-weighted case. The seasonal averaging also improves the match of the precession signal for EPICA Dome C and Dome Fuji. For Vostok, the selected monthly weighting (which was chosen to minimize the RMSE for the total reconstruction) does not result in a better precession signal, but a broader seasonal-mean (such as April-December) matches the proxy signal approximately as well as the annual-mean, as suggested by Supplementary Fig. 9. While age uncertainties certainly still do exist and may

potentially affect the current analysis, the results in this paper generally support the argument that the ice cores are capturing a seasonal, rather than annual, signal.

Supplementary Note 4: Model evidence for linearity of Antarctic response to obliquity

To test the linearity of the Antarctic temperature response to obliquity, we take advantage of a set of 61 experiments run with the HadCM3[30]. These experiments are designed to sample different combinations of orbital, greenhouse gas, and ice sheet forcing in a way that samples the parameter space. CO_2 and astronomical forcing are sampled following a constrained Latin hypercube and ice sheet topography configurations (here represented by a quantity called the "glaciation index", which is 1 for the present-day and 11 for the Last Glacial Maximum) are sampled to browse possible configurations throughout the Quaternary. The output of the experiments are then fitted to a Gaussian process model to estimate the response of the model continuously throughout this forcing space. Full details are given in [30]. Gaussian-process modelling was made using the GP R-package[31].

We concentrate on the response of annual-mean Antarctic surface air temperature, which is here computed as the average over the latitudes 60-90°S. Before using the emulator to investigate the linearity of temperature response to obliquity, we need to verify that the Gaussian Process model captures this response correctly. The procedure relies on a leave-one-out validation technique: the Gaussian process model is trained on all but one experiment, and the excluded experiment is subsequently

predicted with the Gaussian process emulator. The procedure is repeated for all experiments, which yields statistics of prediction errors. For this study, it is particularly important to verify that the prediction errors are independent of obliquity, ice volume, and CO_2 to ensure that we are not missing significant relationships between these inputs and the model outputs.

Prediction errors are plotted in Supplementary Fig. 11, along with the standard deviations of these errors as predicted by the Gaussian process emulator. Visual inspection suggests that there is no relationship between prediction errors and input variables, and this may be further verified using the Hilbert Schmidt Independence Criterion[32,33]. Note that prediction errors are most often smaller than 0.2 °C (with only two errors > 0.4 °C) while Antarctic temperature varies by more than 10 °C throughout the experiment design.

We use the Gaussian process model calibrated on all experiments to predict the relationship between obliquity and temperature over this region, and this is done for different values of glaciation index, CO_2, and precession (Supplementary Fig. 12). As can be seen, the relationship between temperature and obliquity is quite linear. If this result holds true for GFDL CM2.1 and CESM, it suggests that the linear reconstruction approach utilized in this paper is not missing Antarctic responses to obliquity which would be captured by combined-forcing experiments.

Supplementary Note 5: HadCM3 simulations

The HadCM3 simulations mentioned in the main text (shown in Fig. 1c,d) employ

a different experimental design than the CM2.1 and CESM simulations. These simulations are distinct from those mentioned in Supplementary Note 4. Instead of conducting single-forcing experiments for extreme values of the orbital parameters, snapshot simulations were conducted at regular intervals over the past 120 ka, for a total of 62 simulations. Simulations are the same as the ORB-ONLY simulations described in [34], but span the entire last 120 ka. These orbit-only simulations represent the climate response to a wide variety of known orbital combinations, and a multiple linear regression is used to extract the mean climate response to obliquity, different aspects of precession, and eccentricity over the last 120 ka. The design is less straightforward than the approach employed for CM2.1 and CESM, but it does sample the climate response to obliquity over a wide variety of precession and eccentricity combinations, making it a good complement to the CM2.1 and CESM experiments. While the multiple linear regression cannot account for non-linearities in the climate response, the temperature variability which is not accounted for by the regression generally looks like noise. These orbit-only simulations are used to extract the response to obliquity plotted in Fig. 1c,d.

Supplementary References

1. Mantsis, D. F. *et al.* The Response of Large-Scale Circulation to Obliquity-Induced Changes in Meridional Heating Gradients. *J. Clim.* **27,** 5504–5516 (2014).
2. Soden, B. J. *et al.* Quantifying Climate Feedbacks Using Radiative Kernels. *J. Clim.* **21,** 3504–3520 (2008).
3. Erb, M. P., Jackson, C. S. & Broccoli, A. J. Using single-forcing GCM simulations to reconstruct and interpret quaternary climate change. *J. Clim.* **28,** 9746–9767 (2015).
4. Kindler, P. *et al.* Temperature reconstruction from 10 to 120 kyr b2k from the NGRIP ice core. *Clim. Past* **10,** 887–902 (2014).
5. Naafs, B. D. A., Hefter, J., Ferretti, P., Stein, R. & Haug, G. H. Sea surface temperatures did not control the first occurrence of Hudson Strait Heinrich Events during MIS 16. *Paleoceanography* **26,** 1–10 (2011).
6. Kreitz, S. F., Herbert, T. D. & Schuffert, J. D. Alkenone paleothermometry and orbital-scale changes in sea-surface temperature at Site 1020, northern California margin. *Proc. Ocean Drill. Program, Sci. Results* **167,** 153–161 (2000).
7. Rodrigues, T., Voelker, A. H. L., Grimalt, J. O., Abrantes, F. & Naughton, F. Iberian Margin sea surface temperature during MIS 15 to 9 (580-300 ka): Glacial suborbital variability versus interglacial stability. *Paleoceanography* **26,** 1–16 (2011).
8. Herbert, T. D. *et al.* Collapse of the California current during glacial maxima linked

to climate change on land. *Science* **293,** 71–76 (2001).

9. Oppo, D. W. & Sun, Y. Amplitude and timing of sea-surface temperature change in the northern South China Sea: Dynamic link to the East Asian monsoon. *Geology* **33,** 785–788 (2005).

10. Herbert, T. D. *et al.* Tropical ocean temperatures over the past 3.5 million years. *Science* **328,** 1530–4 (2010).

11. Bolliet, T. *et al.* Mindanao Dome variability over the last 160 kyr: Episodic glacial cooling of the West Pacific Warm Pool. *Paleoceanography* **26,** 1–18 (2011).

12. Weldeab, S., Lea, D. W., Schneider, R. R. & Andersen, N. 155,000 years of West African monsoon and ocean thermal evolution. *Science* **316,** 1303–7 (2007).

13. Lea, D. W., Pak, D. K. & Spero, H. J. Climate Impact of Late Quaternary Equatorial Pacific Sea Surface Temperature Variations. *Science* **289,** 1719–1724 (2000).

14. Lea, D. W. *et al.* Paleoclimate history of Galápagos surface waters over the last 135,000 yr. *Quat. Sci. Rev.* **25,** 1152–1167 (2006).

15. Medina-Elizalde, M. & Lea, D. W. The mid-Pleistocene transition in the tropical Pacific. *Science* **310,** 1009–12 (2005).

16. Mohtadi, M. *et al.* Late Pleistocene surface and thermocline conditions of the eastern tropical Indian Ocean. *Quat. Sci. Rev.* **29,** 887–896 (2010).

17. Pahnke, K. & Sachs, J. P. Sea surface temperatures of southern midlatitudes 0-160 kyr B.P. *Paleoceanography* **21,** 1–17 (2006).

18. Groeneveld, J. & Chiessi, C. M. Mg/Ca of Globorotalia inflata as a recorder of

permanent thermocline temperatures in the South Atlantic. *Paleoceanography* **26,** 1–12 (2011).

19. Pelejero, C., Calvo, E., Barrows, T. T., Logan, G. A. & De Deckker, P. South Tasman Sea alkenone palaeothermometry over the last four glacial/interglacial cycles. *Mar. Geol.* **230,** 73–86 (2006).

20. Martinez-Garcia, A. *et al.* Subpolar Link to the Emergence of the Modern Equatorial Pacific Cold Tongue. *Science* **328,** 1550–1553 (2010).

21. Mashiotta, T. A., Lea, D. W. & Spero, H. Glacial–interglacial changes in Subantarctic sea surface temperature and δ18O-water using foraminiferal Mg. *Earth Planet. Sci. Lett.* **170,** 417–432 (1999).

22. Pahnke, K., Zahn, R., Elderfield, H. & Schulz, M. 340,000-Year Centennial-Scale Marine Record of Southern Hemisphere Climatic Oscillation. *Science* **301,** 948–952 (2003).

23. Jouzel, J. *et al.* Orbital and Millennial Antarctic Climate Variability over the Past 800,000 Years. *Science* **317,** 793–796 (2007).

24. Uemura, R. *et al.* Ranges of moisture-source temperature estimated from Antarctic ice cores stable isotope records over glacial-interglacial cycles. *Clim. Past* **8,** 1109–1125 (2012).

25. Parrenin, F. *et al.* Synchronous Change of Atmospheric CO2 and Antarctic Temperature During the Last Deglacial Warming. *Science* **339,** 1060–1063 (2013).

26. Shakun, J. D., Lea, D. W., Lisiecki, L. E. & Raymo, M. E. An 800-kyr record of

global surface ocean δ18O and implications for ice volume-temperature coupling. *Earth Planet. Sci. Lett.* **426,** 58–68 (2015).

27. Dima, I. M. & Wallace, J. M. On the Seasonality of the Hadley Cell. *J. Atmos. Sci.* **60,** 1522–1527 (2003).
28. Soden, B. J. & Held, I. M. An Assessment of Climate Feedbacks in Coupled Ocean–Atmosphere Models. *J. Clim.* **19,** 3354–3360 (2006).
29. MacKay, S. L. & Marchant, D. R. Obliquity-paced climate change recorded in Antarctic debris-covered glaciers. *Nat. Commun.* **8,** 1–12 (2017).
30. Araya-Melo, P. A., Crucifix, M. & Bounceur, N. Global sensitivity analysis of Indian Monsoon during the Pleistocene. *Clim. Past* **11,** 45–61 (2015).
31. Crucifix, M. package ‘GP’, R-package, version 0.1.7. (2016).
32. Gretton, A. *et al.* A kernel statistical test of independence. *Neural Inf. Process. Syst.* 585–592 (2008).
33. Pfister, J. & Pfister, N. package ‘dHSIC’, R-Package, March 2. (2016).
34. Singarayer, J. S., Valdes, P. J., Friedlingstein, P., Nelson, S. & Beerling, D. J. Late Holocene methane rise caused by orbitally controlled increase in tropical sources. *Nature* **470,** 82–85 (2011).
